# Necessity and influencing factors for integrating oral health in cancer care for older people: a narrative review

**DOI:** 10.1007/s00520-024-08632-x

**Published:** 2024-06-26

**Authors:** Shalya Anand, Anita Visser, Joel B. Epstein, Djenana Jalovcic

**Affiliations:** 1 Department for Global Health and Rehabilitation, Western Norway University of Applied Sciences, Bergen, Norway; 2 Max Planck Institute for Human Development, Center for Adaptive Rationality, Berlin, Germany; 3 Department of Gerodontology, Center for Dentistry and Oral Hygiene, University of Groningen, University Medical Center Groningen, Groningen, The Netherlands; 4 Department of Gerodontology, College of Dental Sciences, Radboud University Nijmegen Medical Centre, Nijmegen, The Netherlands; 5 Dental Oncology Services, City of Hope National Cancer Center, Duarte, CA USA; 6 Cedars-Sinai Health System, Los Angeles, CA USA

**Keywords:** Integrated oral healthcare, Healthy aging, Oral complications of cancer therapies, Cancer care for older people/geriatric oncological care, Integrated cancer care, Interprofessional collaboration and education, Oral health literacy, Multidisciplinary teams

## Abstract

**Purpose:**

The number of older people with poor oral health diagnosed with cancer is increasing rapidly. However, integration of oral health in cancer care for older people to prevent or minimize oral health complications of cancer treatments is uncommon, except in head and neck oncology. The aim of this review is to describe the need, role of, and factors influencing the integration of oral health(care) into the treatment of older people with cancer*.*

**Methods:**

MEDLINE, CINAHL, PubMed, Scopus, and Web of Science databases were searched for papers published in the last 10 years that focus on oral health in older people diagnosed with cancer, the impact of oral health on cancer therapy, and integrated oral health in cancer treatment.

**Results:**

From 523 related papers, 68 publications were included and summarized as follows: (1) oral complications associated with cancer therapies, (2) the need for oral healthcare in older people with cancer, (3) the role of integration of oral health in cancer care, and (4) influencing factors such as ageism, interprofessional education and collaborations, oral healthcare workforce, oral health literacy, and financial considerations.

**Conclusion:**

Integration of oral healthcare is highly recommended for the overall well-being of older people with cancer to prevent, minimize, and manage complications in cancer treatment. However, oral healthcare has not been integrated in cancer care yet, except for head and neck cancers. This review identified a notable gap in the literature, highlighting the need for research on integration of oral healthcare in geriatric oncology.

## Introduction

Older people will constitute 22% of the global population by 2050 necessitating a recognition of healthy aging in health policies which is not merely an absence of disease, but a process of developing and maintaining the functional ability to enable well-being, happiness, and fulfillment in older age [1, 2]. Oral health is necessary to maintain functional and social capabilities for the overall well-being of older persons [3, 4]. Additionally, age is a significant risk factor for cancer diagnosis, as 68% of the 19.3 million new cancer cases and 70% of the 10 million cancer deaths in 2020 were among older people aged > 65 years [5, 6]. Advances in cancer treatments prolong life, however, age-related health issues and comorbidities in older people such as neurodegenerative diseases, chronic obstructive pulmonary disease, diabetes, polypharmacy, weakened immune systems, coupled with poor oral health (chronic inflammation, caries, oral pain, chewing problems, mucositis, xerostomia, dysphagia, etc.), can be considered as complicating health factors [5, 7–10]. These factors can negatively impact cancer treatment outcomes with significant morbidity and mortality, requiring interventions to improve well-being and treatment outcomes for older people with cancer.

Literature shows a worldwide increase in multidisciplinary oncology teams comprising of oncologists, medical specialists, geriatricians, primary care practitioners, and nursing staff who provide comprehensive “geriacentric” oncology care in hospitals with oncology care centers [11]. However, apart from head and neck cancers, oral health professionals are not included in such teams. Furthermore, there is limited research that suggests the need and role of the inclusion of an oral healthcare team, to screen, prevent, manage, and treat the oral complications of cytotoxic cancer therapies for cancer in older people [10]. The aim of this review is to describe the need, role of, and factors influencing the integration of oral health(care) into the treatment of older people with cancer*.*

## Methods

A narrative review was chosen to synthesize and summarize the literature, and to formulate arguments for integrating oral healthcare in cancer care, except for head and neck cancers where oral health is recognized as a part of that care. A three-step framework of literature search and screening, data extraction, and analysis was performed. The Participant-Concept-Context framework was used to develop the search strategy: Participants: Older people diagnosed with cancer; Concept: (Integrated) oral health(care); Context: Cancer treatment outcome and Oral complications during or after cancer therapies.

The search performed by the principal author and a research librarian included MEDLINE, CINAHL, PubMed, Scopus, and Web of Science databases. The search strategy utilized Medical Subject heading [MeSH] terms and keywords being aged, elderly, or older people*, neoplas* or cancer*, [oral* or dental*] NEAR/O [health care or complicat*], integrat* NEAR/O care, [interdisciplinar* or transdisciplinar* or support* or palliativ*] NEAR/O care. To ensure that the age classification and inclusion criteria remained unambiguous, articles for older people above the age of 60 were included. Terms such as “older people,” “older people with oral health disease,” and “older people with cancer” are used to avoid discrimination and negative stereotypes for older people [12]. Articles reporting on oral healthcare in young people with cancer were excluded.

The search included peer-reviewed articles using qualitative, quantitative, or mixed methods, reviews, policy documents, and commentaries, from oncology, dentistry, nursing, geriatrics, and healthy aging, published in English between January 2012 and November 2023. Title and abstracts were read to select the relevant articles. References were manually checked to identify relevant articles that were not part of the search results. A quality assessment was not performed considering this is a narrative review.

## Results

**Fig. 1:**
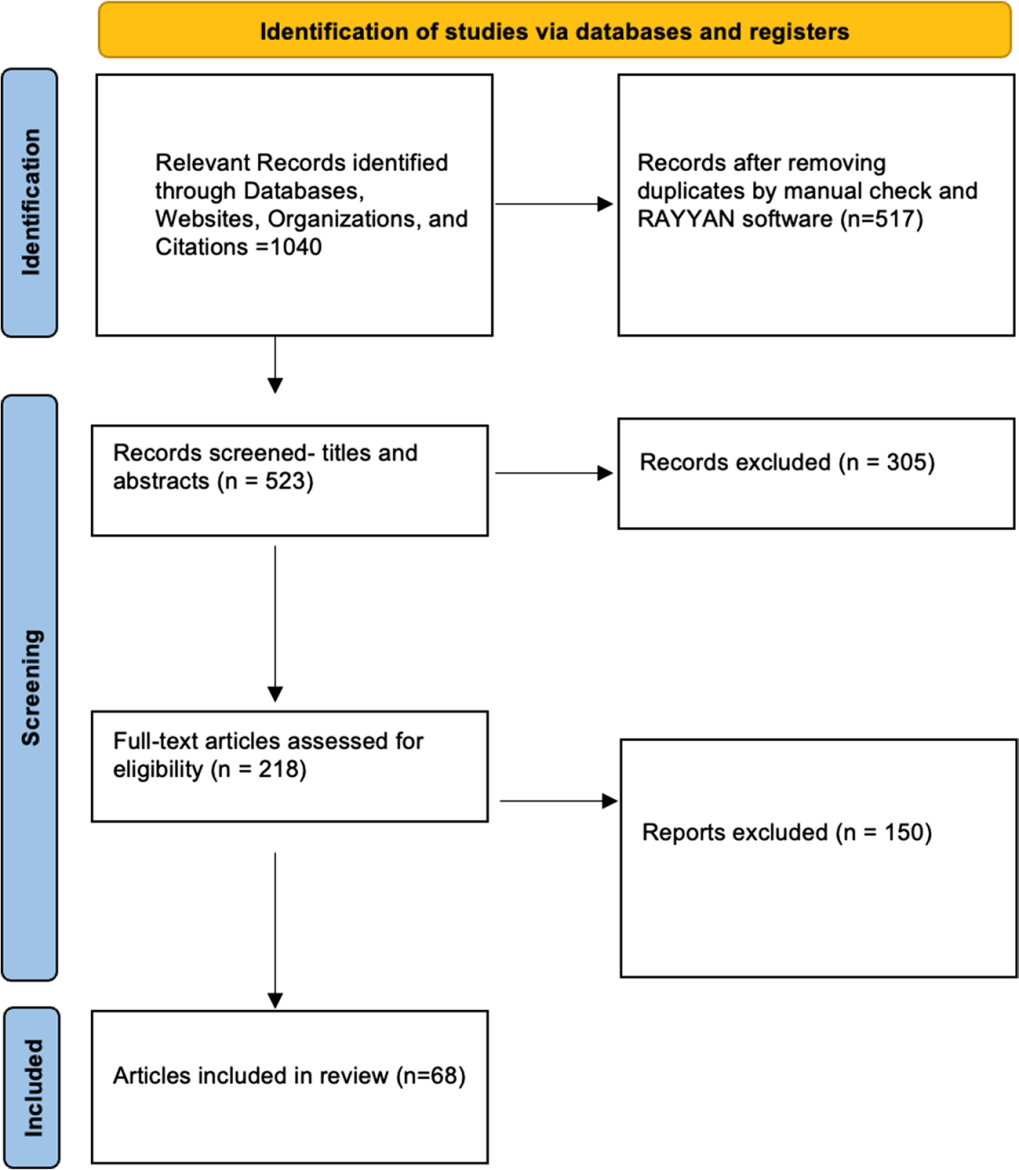
The PRISMA flow chart

The initial search resulted in 1040 articles. After removal of duplicates and manual check, titles and abstracts of 523 related articles were screened resulting in 216 articles for full-text screening, out of which 68 articles were selected for this review (Fig. 1).

**Fig. 2:**
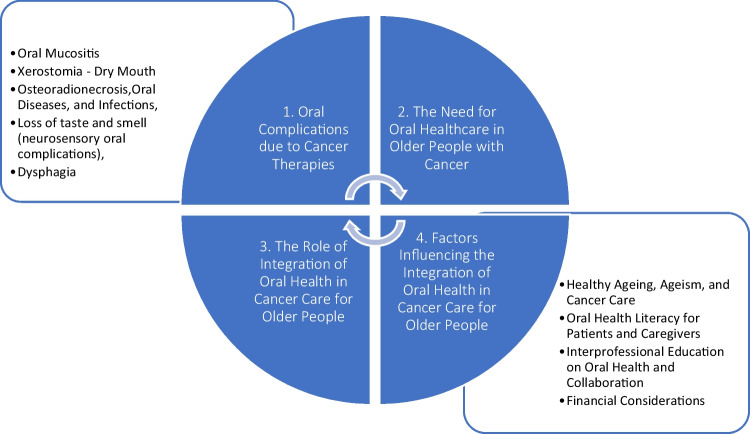
Flow chart: summary of results

The results are organized in four sections (Fig. 2): (1) oral complications due to cancer therapies; (2) the need for oral healthcare in older people with cancer; (3) the role of integration of oral health in cancer care, and (4) factors influencing the integration of oral health in cancer care for older people.

- 1. Oral complications due to cancer therapies

Studies prove that life-prolonging cancer treatments such as chemotherapy, radiotherapy, hematopoietic stem cell transplantation, supportive anti-resorptive therapies, and targeted and immune therapies have cytotoxic effects on oral tissues resulting in various oral complications [9, 10, 13–20]. The most common oral complications are summarized in Table 1.

- 2. The need for oral healthcare in older people with cancer

**Table 1:** Oral complications due to cancer therapies

Oral complications	Details
Oral mucositis [10, 14]	A common condition characterized by inflammation and ulceration of the mucous membranes in the mouth, leading to severe pain and discomfort in normal oral functions (chewing, swallowing, speaking, and maintaining oral hygiene). It is considered an acute toxicity in patients receiving radiation, chemotherapy, combination therapies, and hematopoietic cell transplantation. Mucosal ulceration can result in malnutrition, increased risk of infection, use of opioids, and hospitalization, and may potentially limit cancer therapy
Xerostomia–dry mouth [10, 15]	Almost 50% of cancer patients undergoing treatment present acute side effects such as thickened saliva and decreased saliva production causing xerostomia. The absence of saliva in the mouth acts as a precursor to an increased incidence of caries, periodontal disease, fungal infections (candida), and neuropathic pains. The various oral diseases, including oral ulcerations, infections, tumor surgery, chemoradiotherapy, and tooth extractions, may result in increased functional impairments in older patients with cancer
Osteoradionecrosis, oral diseases, and infections [9, 16–18]	Although primarily occurring as the delayed and chronic adverse effects of radiotherapy for head and neck cancers, chemotherapy with bone-modifying anti-resorptive agents and anti-angiogenic agents, surgical treatment for tumors, and chemoradiotherapy can impact both hard and soft oral tissues. These may result in oral ulcerations/mucositis, acute necrotizing ulcerative stomatitis, and fungal infections such as oral thrush or candidiasis. Old age, treatment duration, pre-existing oral diseases (caries, periodontal diseases, missing teeth), sharp teeth edges, ulcers due to misfitting of dentures, and habits like smoking and alcohol increase susceptibility to bone necrosis and infections
Loss of taste and smell (neurosensory oral complications), dysphagia [9, 17–20]	Chemotherapy with drugs such as docetaxel, anthracyclines, and paclitaxel, which are described for breast, colorectal, and female reproductive cancers may lead to dysgeusia and dysosmia, i.e., taste and smell alterations. Additionally, difficulty in swallowing (dysphagia) due to surgical therapy can affect the nutritional status, well-being, quality of life, and survivorship. Poor throat muscle control coupled with oral bacteria and oral debris from a diseased infected mouth may be transferred to the lungs and cause aspiration pneumonia, with increased mortality

Older people with comorbidities, polypharmacy, and age-related health problems have a high risk of developing oral diseases especially when cancer is among individual’s health problems. Various acute and chronic oral complications due to cancer diagnosis and associated treatments can occur [10]. Evidence indicates that older people are prone to oral frailty, defined as a set of phenomena and processes that result in age-related changes in various oral conditions, e.g., the number of remaining teeth, oral hygiene, oral dysfunction, saliva function, decreased interest in oral health, and reduced physical and mental reserve capacity for regular oral healthcare [8, 10]. Cancer treatments worsen oral frailty increasing oral diseases and infections, such as caries, periodontal diseases, edentulism, and dry mouth, acting as a precursor to calorie/nutrient deficiency, pain, infections, difficulty in speaking, loss of confidence, and social isolation, resulting in limited oral functional ability, orofacial pain, poor general physical health, psychological status, and functional capacity in older people with cancer. This can result in malnutrition, which in turn negatively impacts the immune system, which is an important process in fighting cancer, further influencing the treatment outcome of older people with cancer [3, 8, 10, 21–26].

- 3. The role of integration of oral health in cancer care for older people

Academic literature confirms that cancer treatments affect the oral tissues [8, 10, 13–15, 17–20]. Studies suggest that the integration of oral healthcare in the cancer care pathway improve the treatment outcomes of cancer therapies and the overall health and well-being of patients with cancer [10, 27–30]. The call for early diagnosis, prevention, and treatment of oral health problems in patients undergoing cancer treatment through the integration of oral health assessment at the intake procedure for cancer patients is not new. It was first recommended by the National Institutes of Health in 1989, which believed that dentists were well positioned for effective minimization, treatment, and management of oral complications and to prevent adverse health outcomes [28–30]. Studies recommend appropriate oral screening, prevention, and management strategies, with an oral health examination and counseling for basic oral care, before and every 3 months during the first year of oncological treatment, with biannual follow-up maintenance visits for long-term survivorship and quality of life for patients with cancer [10, 31]. Research evidence shows that timely intervention through basic oral healthcare, with professional oral prophylaxis considered the gold standard of care, can reduce the total number of infective bacteria by 10–100-fold, and reduce oral complications due to cancer treatments [9, 20]. Studies show that exacerbation of acute and chronic oral complications of cancer therapies can be associated with increased fatigue, calorie and nutritional deficiencies, hospitalization, severe pain, and life-threatening systemic infectious complications. In older people with pre-existing conditions, it may lead to premature cessation of cancer treatments, increasing the chances of morbidity and mortality [8–10, 19, 20, 22, 28, 29, 32–36]. Oral health assessment, oral hygiene treatments, and advice for regular basic oral care included in the care for older people may result in increased ability to eat, speak, and swallow, improved nutritional status, patient satisfaction, treatment compliance, enhanced self-esteem, and well-being [10, 27].

- 4. Factors influencing the integration of oral health in cancer care for older people

While several studies have examined the benefits of the integration of oral healthcare professionals in head and neck cancer multidisciplinary teams, limited evidence exists for such collaborations for older people undergoing treatment for cancer at other sites [10, 14, 16, 20, 21, 27, 36–38]. Literature confirms and recognizes the importance of appropriate clinical practice guidelines through timely oral healthcare to manage and minimize the impact of oral complications due to cancer treatments [15, 18, 28, 30, 35, 39]. Literature that is included in this narrative review highlights the following key influencing factors that may be affecting the integration of oral healthcare into cancer care for older people: (a) healthy aging, ageism, and cancer care; (b) oral health literacy for patients and caregivers; (c) lack of interprofessional education on oral health and lack of collaboration between oral healthcare professionals and oncology specialists; and (d) financial considerations.

- a) Healthy aging, ageism, and cancer care

Older people have unique oral healthcare needs to achieve healthy aging which involves functional and psychosocial capabilities to enable well-being, happiness, satisfaction, and fulfillment [1, 2]. Healthy aging is strongly associated with good oral health [3]. Oral health is essential for social interaction, communication and speech, taste, touch, nutrition (taste, chewing, swallowing), and to convey a range of emotions through facial expressionswithout pain, and disease of the craniofacial complex [4, 40–42]. Literature confirms that oral health is essential for overall well-being and quality of life for older people [3, 4]. It is of particular importance to older people being treated for cancer but there is limited research in this field till date [10]. The literature suggests that ageism, or discrimination against individuals based on their age, may lead to reduced quality of care due to underassessment of symptoms, poor accessibility to supportive and palliative care, and exclusion of older adults with cancer from decision-making processes [1, 23, 43, 44]. Moreover, studies show that oral healthcare has been viewed as an individual concern and not included in universal health coverage and global public health programs [25, 45–47]. Recent developments emphasize the growing focus of the current political, policy, and clinical leadership toward healthy aging [1, 5]. However, there is a notable absence of emphasis on oral health within the agenda of the United Nations Decade of Healthy Aging toward Integrated Care for Older People (ICOPE), which strives for “person-centered and coordinated care” to “deliver person-centered, integrated care and primary health services responsive to older people” [48].

- b) Oral health literacy for patients and caregivers.

Integrating oral health in cancer care for older people can be complicated due to the presence of comorbidities, polypharmacy, cognitive decline, limited caregiver support, social isolation, and financial and functional dependency [5, 7, 35, 43, 49, 50]. Furthermore, poor oral health literacy can result in a lack of understanding regarding the importance of oral care, leading to inadequate home oral hygiene care and delayed visits to healthcare providers, increasing healthcare and societal costs of cancer treatment [5, 10, 31, 51]. Studies reporting the perceptions of patients and caregivers recognize the impact of socio-demographic status, cultural influences, and education to cater to urgent medical needs compared to the oral healthcare needs of older people [10, 52]. Literature indicates that oral health education can be facilitated as a part of cancer care counseling, for improved provision of oral care through preventive measures such as fluoride treatments, and timely prescription of indicated gels, rinses, and medication unique to the patient’s oral complications and oral status [17, 18, 20, 29, 31]. Medical advice supporting the need for oral evaluation and care is important in emphasizing the need to patients and to reinforce compliance with oral care and preventive measures. Though no studies exist specifically for older people with cancer, studies emphasizing a patient-centered approach through innovative and customized oral hygiene care via home visits, telecare, and modifications of toothbrushes, written agendas with easy-to-understand instructions, and the use of apps on mobile phones, could ensure consistent and coordinated care across the cancer treatment trajectory [9, 10, 13, 17, 18, 20, 28–30, 39, 51–55].

- c) Interprofessional education on oral health and collaboration between oral healthcare professionals and oncology specialists

Collaborative efforts through multidisciplinary teams comprising oncologists, geriatricians, primary care practitioners, nursing, and supportive care staff, provide comprehensive “geriacentric” oncology care to the growing number of older people with a cancer diagnosis [37]. However, this review found only one study that focused on the oral healthcare needs of older people with cancer [10]. Older persons tend to trust their physicians and oncologists more than dentists for health communication about their oral complications and their recommendations and reinforcement are important for increased patient compliance [11]. Literature suggests that the barriers to the integration of oral healthcare in geriatric oncological care include a lack of focus on oral health-related needs in interdisciplinary collaborations through limited inclusion of patients, supportive care staff, and caregivers for patient-centered perspectives and information in multidisciplinary decision-making teams, and an overall lack of interprofessional education and subsequent collaborations [5, 10, 11, 17, 21, 22, 26, 34, 37, 38, 45, 54, 56–58]. The nursing staff in oncology teams with a high workload, and limited oral health education and training, usually face difficulty in rendering individualized oral care to hospitalized older people with cancer [34, 53]. There is evidence that the oral health literacy of hospital care managers and administrators affects the promotion of oral care educational programs and appropriate staffing of trained oral care assistants and nursing staff [34, 49]. Additionally, there is a significant lack of expert oral healthcare providers for older people, and more so for patients undergoing different cancer treatments [10, 59]. Studies show that limited interprofessional knowledge is a barrier for dentists and recommend capacity building for the increased number of skilled workforces to cater to the needs of older people with cancer [21, 25, 34, 38, 57]. The literature recommends innovative approaches to support geriatric dental care, e.g., adaptation of dental operatories for wheelchair accessibility, home visits, teledentistry, skill mix, minimally invasive dentistry, digital technology, electronic patient records, and use of digital tools and smart applications [38, 55].

- d) Financial considerations

Evidence suggests that oral diseases are usually considered an optional treatment for older people due to the ongoing financial burden of treatment of cancer and/or other systemic disease. Suboptimal oral health may be considered acceptable as part of the aging process, based on socio-economic status and oral health literacy [60–63]. Out-of-pocket expenditure for the high costs of dental treatments, limited/non-existent dental insurance plans, lack of government support, and non-existent/limited funding for global oral healthcare prevention programs renders oral care unaffordable and thus neglected till urgent and unavoidable [24, 61]. However, various studies suggest that the long-term effect of avoiding oral health treatments has higher healthcare and societal costs due to exacerbation of systemic diseases, increased rate of hospitalization due to pain, malnutrition, higher morbidity and mortality, and productivity costs of patients and caregivers [22, 23, 52, 60, 62–64]. The inclusion of oral healthcare as “best-buys” in the global agenda for non-communicable diseases by the World Health Organization aims to provide an incentive for the integration of oral health in cancer care [50, 65]

## Discussion

This narrative review aims to describe the need, role of, and factors influencing the integration of oral health(care) into the treatment of older people with cancer*.* It includes literature to focus on the importance of oral health as a part of the geriatric cancer care pathway. Its findings emphasize the increasing numbers of cancers in older age and the importance of good oral health for healthy aging especially in the presence of comorbidities and the interactions between oral and general health. A “geriacentric” strategy for equitable access through acknowledging the disparities and developing innovative approaches, with a focus on health and (oral) care, rather than disease and cure, could enable older people to maintain a dignified, independent life, with a focus on capacities such as happiness, well-being, and fulfillment [1, 2].

This review establishes the complexity of cancer care and the oral complications due to diverse cancer treatments. It reveals the effect of cancer treatment on oral health and the profound impact of poor oral health on treatment outcomes and complications during cancer treatment of older people [8–10, 13–22, 45]. Concurrently, the findings confirm that good oral health is essential for the general well-being of older people through the maintenance of nutrition, lifestyle, and socio-psychological well-being [3, 8, 10, 21–26, 45, 40–42]. Integrating oral healthcare in cancer care, as suggested by multiple studies not limited to older people, has been shown to enhance the overall well-being, quality of life, and survivorship of patients with cancer, due to improved ability to eat, speak, and swallow, healthier nutritional status, and increased compliance with cancer treatments [10, 27–30]. Various studies emphasize the importance of timely oral healthcare to prevent malnutrition, pain, infection, speech difficulties, and loss of self-confidence, social isolation, and poor general and oral health-related quality of life in older people [8–10, 19, 20, 22, 28, 29, 32–36, 40–42]. This review found studies suggesting that every cancer patient should be referred for an oral health examination and counseling, for basic home care oral hygiene treatments before and every 3 months in the first year of oncological treatment, with biannual follow-up maintenance visits to reduce the bacterial contamination of the oral cavity, thereby managing, and minimizing certain oral complications of cancer treatments [10, 20, 27–31].

Although oral complications of life-prolonging cancer therapies are inevitable, measures can be undertaken to minimize their impact on the functional and psychosocial well-being of older people with cancer, through focus on the oral cavity as a pathway for general health and nutrition. Oral frailty, pre-existing diseases, and their treatments make older people diagnosed with cancer respond differently to oral complications of their cancer therapies, due to their heterogeneous and varied multimorbidity status, with individual unique needs, necessitating expert oral care depending on the diagnosis and required dental and cancer treatments [8, 10, 21, 22, 45]. The inability to eat, chew, and swallow may be compounded by the inability to wear dentures/prostheses, resulting in pain, infections, malnutrition, and an increased rate of hospitalization [10, 21, 45].

This review identifies ageism and inequalities in cancer care, with scarcity of articles with evidence-based recommendations for the integration of oral healthcare in cancer care for older people [1, 10, 21, 23, 37, 43]. This gap could be the reason for the lack of clinical guidelines for timely assessments, investigations, and management through specific recommendations for diagnosis, prevention, and management of oral conditions unique to oncology care and at increased impact in older people. This review establishes that existing comprehensive geriatric assessment and screening tools in multidisciplinary teams could be integral in identifying, evaluating, and triaging patients for dental referrals and education. Depending on the disease phase, consequent treatment, and existing oral health, resource-intensive interventions may be designed.

However, studies confirmed that unless referred to or advised by the oncologist, patients with cancer are less likely to get an oral health check-up [17, 18, 20, 29, 31]. This review identifies poor interprofessional education as one of the prime reasons for limited interprofessional collaborations. Various studies discuss the siloed nature of healthcare systems, where oncology is a limited component of dental undergraduate and graduate education and vice-versa. The dental community is generally not prepared for oral care of medically complex cancer patients, leading to a growing demand for an oral healthcare workforce trained in gerodontology (oral healthcare for older people), and geriatric oncology to manage the unique oral healthcare challenges and needs of older people with cancer. This narrative review highlights the need for a trained workforce and appropriate standardized guidelines for the nursing staff in clinics, hospitals, nursing care homes, and old age homes. Promoting interprofessional education is necessary to emphasize the need, and importance, and facilitate the successful integration of oral healthcare in cancer care for older people with cancer. It shall facilitate patient-centered advice according to the pre-existing oral conditions and prosthesis, the importance of maintaining oral hygiene regularly, regular checkups and preventive treatments, and timely prescription of appropriate indicated gels, rinses, and medication, for successful cancer treatment outcomes [5, 10, 11, 17, 21, 22, 26, 34, 37, 38, 45, 54, 56–59].

Evidence with the use of artificial intelligence in dentistry may help in understanding the unique needs and characteristics of patients by using predictive analytics for early detection of oral diseases by identifying high-risk individuals, and the use of teledentistry for remote consultations. Studies emphasize the use of digital and technological advances for data sharing, flexible clinical trial designs for toxicity specific to older people, comprehensive geriatric assessments, and screening tools in multidisciplinary teams to identify, evaluate, and refer patients for dental care and education, encouraging appropriate interventions for older people [9, 10, 13, 17, 18, 20, 28–30, 39, 51–55]. An adaptation of dental operatories for wheelchair accessibility, minimally invasive dentistry, and individualized oral care products such as toothbrushes with modified grip and ultra-soft bristles, can further support dental care for older people with cancer [38, 55].

Furthermore, this review establishes the need to empower older people with cancer and caregivers to become active partners in oncological care. Healthcare providers, patients, and caregivers need to share responsibility and play a significant role in sharing information, advocating, and empowering behavioral changes toward regular and timely oral hygiene maintenance, greater patient engagement, and better health outcomes by minimizing oral complications. Oral health literacy through cancer counseling sessions could motivate the patients and caregivers for timely detection, referrals, and interventions, through integration of oral and public health promotion, information, and service strategies [17, 18, 20, 29, 31, 51, 52].

Included studies affirm that oral healthcare is generally considered optional care for older people, whereas suboptimal oral health with missing teeth and oral disease is considered common. Although the financial burden of private dental care renders oral healthcare avoidable, studies prove that poor oral health results can increase higher healthcare and societal costs in cancer care [22–24, 52, 60–64]. The Federal Medicare proposal by the United States Central Management Services, to include medically necessary oral/dental care in the Medicare system in 2024, is a positive step toward the recognized acknowledgment of the importance of oral care in the realm of oncology care [66]. Advocacy for integrating medical and oral health insurance coverage for cancer care through innovative financial approaches may be supported by the inclusion of oral healthcare in the global agenda for non-communicable diseases by the World Health Organization [50].

## Strengths and limitations

This review aims to serve as a resource to inform decision-makers and stakeholders in geriatric cancer care through the identification of research gaps with a comprehensive compilation of evidence. To minimize the researcher’s citation bias and the methodological limitations, relevant evidence was selected with justice and purpose, acknowledging the inability to comprehensively cover factors including socio-economic status, contextual and geographical differences in healthcare systems, age-related differences, and types of cancer treatments [67, 68].

## Recommendations

This review recommends addressing the identified gaps in epidemiological data on existing standards of care, reforms in clinical dental education, interprofessional collaborations to define treatment guidelines, and the evaluation of the impact of interventions and clinical trials that integrate oral healthcare in cancer care for older people. Evidence from validated shared tools such as clinical practice guidelines, measurement of patient-reported outcomes, and economic evaluation studies can inform policymakers for decision-making in healthcare.

## Conclusion

Integration of oral healthcare is highly recommended for the overall well-being of older people with cancer to prevent, minimize, and manage complications in cancer treatment. However, oral healthcare has not been integrated in cancer care except for head and neck cancer patients. This review identified a notable gap in the literature, highlighting the need for research on integration of oral healthcare in geriatric oncology.
